# Prognostic impact of the atherogenic index of plasma in type 2 diabetes mellitus patients with acute coronary syndrome undergoing percutaneous coronary intervention

**DOI:** 10.1186/s12944-020-01418-0

**Published:** 2020-11-16

**Authors:** Xiaoteng Ma, Yan Sun, Yujing Cheng, Hua Shen, Fei Gao, Jing Qi, Lixia Yang, Zhijian Wang, Dongmei Shi, Yuyang Liu, Xiaoli Liu, Yujie Zhou

**Affiliations:** Department of Cardiology, Beijing Anzhen Hospital, Capital Medical University, Beijing Institute of Heart Lung and Blood Vessel Disease, Beijing Key Laboratory of Precision Medicine of Coronary Atherosclerotic Disease, Clinical Center for Coronary Heart Disease, Capital Medical University, Beijing, 100029 China

**Keywords:** Atherogenic index of plasma, Type 2 diabetes mellitus, Acute coronary syndrome, Percutaneous coronary intervention, Adverse cardiovascular events

## Abstract

**Background:**

The association of the atherogenic index of plasma (AIP), an emerging lipid index that can predict the risk for cardiovascular disease, with adverse outcomes in type 2 diabetes mellitus (T2DM) patients with acute coronary syndrome (ACS) undergoing percutaneous coronary intervention (PCI) has not been determined. Therefore, the aim of this study was to investigate whether the AIP could independently predict adverse cardiovascular events in T2DM patients with ACS undergoing PCI.

**Methods:**

This study was a retrospective analysis of a single-centre prospective registry involving 826 consecutive T2DM patients who underwent primary or elective PCI for ACS from June 2016 to November 2017. This study ultimately included 798 patients (age, 61 ± 10 years; male, 72.7%). The AIP was calculated as the base 10 logarithm of the ratio of the plasma concentration of triglycerides to high-density lipoprotein-cholesterol (HDL-C). All the patients were divided into 4 groups based on the AIP quartiles. The primary endpoint was a composite of death from any cause, non-fatal spontaneous myocardial infarction (MI), non-fatal ischaemic stroke, and unplanned repeat revascularization. The key secondary endpoint was a composite of cardiovascular death, non-fatal MI, and non-fatal ischaemic stroke.

**Results:**

During a median follow-up period of 927 days, 198 patients developed at least one event. An unadjusted Kaplan-Meier analysis showed that the incidence of the primary endpoint increased gradually with rising AIP quartiles (log-rank test, *P* = 0.001). A multivariate Cox proportional hazards analysis revealed that compared with the lowest AIP quartile, the top AIP quartile was associated with significantly increased risk for the primary and key secondary endpoints (hazard ratio [HR]: 2.249, 95% confidence interval [CI]: 1.438 to 3.517, *P* < 0.001; and HR: 2.571, 95% CI: 1.027 to 6.440, *P* = 0.044, respectively).

**Conclusions:**

A higher AI*P* value on admission was independently and strongly associated with adverse cardiovascular events in T2DM patients with ACS undergoing PCI.

## Background

Type 2 diabetes mellitus (T2DM) has been established as an important risk factor for adverse outcomes in patients with acute coronary syndrome (ACS) [1–3]; this phenomenon may be due to the rapid development of atherosclerosis related to T2DM [4–7]. T2DM patients with ACS remain at a very high residual risk of adverse cardiovascular events and mortality despite receiving percutaneous coronary intervention (PCI) and guideline-recommended first-line medication therapies [8–11]. Therefore, further risk assessment of such patients may help better determine prognosis and guide better medical management.

Dyslipidaemia is a common comorbidity in T2DM and ACS patients. The dyslipidaemic pattern in diabetic patients [mainly characterized by high levels of fasting and postprandial triglyceride-rich lipoproteins, reduced high-density lipoprotein-cholesterol (HDL-C), normal or slightly elevated low-density lipoprotein-cholesterol (LDL-C), and increased number of small dense LDL-C particles] is somewhat different from that in non-diabetic patients [12]. The atherogenic index of plasma (AIP), readily calculated from the lipid profile as log_10_ (triglycerides/HDL-C), is considered an alternative and simple marker of plasma atherogenicity based on an observed significant, positive relationship between the AIP and cholesterol esterification rates in apoB-lipoprotein-depleted plasma (FER (HDL)), very-low-density lipoprotein particle size, remnant lipoprotein particle cholesterol, and LDL density and the inverse correlation of the AIP with particle sizes of HDL and LDL [13–19]. As a consequence, AIP can be presumed to reflect the lipid characteristics of diabetic patients. Recently, AIP has been demonstrated to be associated with cardiovascular outcomes in the general population and different patient groups [20–31]. However, the prognostic impact of AIP on admission among T2DM patients with ACS undergoing PCI has not been exclusively studied. AIP on admission is hypothesized to be an independent predictor of adverse cardiovascular events in T2DM and ACS patients who underwent PCI.

## Methods

### Study population

This study was a retrospective analysis of a single-centre prospective registry involving 826 consecutive T2DM patients who underwent primary or elective PCI for ACS from June 2016 to November 2017 [32]. The inclusion criteria included diagnosis of ACS according to the American College of Cardiology/American Heart Association guidelines [33, 34] and T2DM according to American Diabetes Association guidelines [35], as well as treatment with primary or elective PCI. For the purpose of this study, patients with prior coronary artery bypass grafting, left ventricular ejection fraction (LVEF) < 30%, cardiogenic shock, and renal failure requiring haemodialysis were excluded. Cardiogenic shock was defined as a condition in which systolic blood pressure remains below 90 mmHg despite adequate blood perfusion, accompanied by clinical signs or laboratory parameters suggestive of hypoperfusion, or in which systolic blood pressure is maintained above 90 mmHg with positive inotropic agents and/or mechanical circulatory support. Renal failure requiring haemodialysis was defined as having a creatinine clearance of less than 15 ml/min or being on haemodialysis. Three patients were also excluded due to lack of follow-up data. A total of 798 patients were eventually included in the present analysis. None of the patients received medications specifically designed to raise HDL-C or lower triglyceride levels, such as niacin, fibrates, and omega-3 fatty acids, before admission or at discharge. For statins, all patients were given either atorvastatin or rosuvastatin, and the vast majority took regular doses of atorvastatin or rosuvastatin (i.e., atorvastatin 20 mg or rosuvastatin 10 mg).

### Measurement

The fasting plasma glucose (FPG), total cholesterol, HDL-C, and triglyceride levels after admission were measured in the central laboratory of Beijing Anzhen Hospital. The Friedewald equation was used to calculate the LDL-C level. Dyslipidaemia was defined as fasting total cholesterol > 5.17 mmol/L, LDL-C > 3.36 mmol/L, triglycerides > 1.69 mmol/L, HDL-C < 1.03 mmol/L, and/or chronic use of lipid-lowering drugs. The AIP was calculated as the log_10_ of the ratio of the plasma concentration of triglycerides to HDL-C [13]. Moreover, demographics, medical history and medication history data for all patients were collected using standard questionnaires.

### Follow-up and endpoints

The follow-up time points were 1 month and every 6 months after hospital discharge. Trained personnel who were not aware of patients’ baseline data obtained information about adverse events via telephone contact with patients or their family members using standardized questionnaires; adverse events were identified by carefully reviewing the corresponding medical records. The primary endpoint was a composite of death from any cause, non-fatal spontaneous myocardial infarction (MI), non-fatal ischaemic stroke, and unplanned repeat revascularization. The key secondary endpoint was a composite of cardiovascular death, non-fatal MI, and non-fatal ischaemic stroke. Adverse events were defined in accordance with previous publications [32, 36]. The follow-up period ended in November 2019.

### Statistical analysis

Power analyses to assess the required sample size were not performed since the present study is a retrospective analysis of a single-centre prospective registry, and currently, there is no literature on longitudinal analyses of the prognostic value of the AIP in a similar study population; therefore, adequate estimation of power is not possible.

All patients were stratified into 4 groups (Q1 [AIP ≤0.0147], Q2 [0.0147 < AIP ≤0.1850], Q3 [0.1850 < AIP ≤0.3517] and Q4 [AIP > 0.3517]) according to the AIP quartiles. Categorical variables were expressed as frequencies and percentages. Chi-squared test or Fisher’s exact test was applied to determine the statistical significance of the differences in categorical variables between groups. Continuous variables with parametric distributions were expressed as the means ± standard deviations, and those with non-parametric distributions were expressed as medians and interquartile ranges. Unpaired t-test or Mann-Whitney U test and analysis of variance or Kruskal-Wallis H test were used to estimate the statistical significance of differences in continuous variables between groups. Kaplan-Meier curves and Cox proportional hazards models were used to conduct survival analyses of the primary and key secondary endpoints. The log-rank test was used to evaluate the differences between Kaplan-Meier estimates. Cox proportional hazards analysis results are presented using hazard ratios (HRs) with 95% confidence intervals (CIs). Variables of statistical significance in the univariate analysis as well as clinical importance were included in the multivariate Cox proportional hazards model. In the multivariate model for the primary endpoint, the following confounding variables were chosen: age (continuous, per 1-year increase), body mass index (BMI) (continuous, per 1-unit increase), hypertension (with or without), previous MI (with or without), past PCI (with or without), peripheral artery disease (with or without), cardiac failure (with or without), LVEF (continuous, per 1% increase), serum creatinine (continuous, per 1-unit increase), LDL-C (continuous, per 1-unit increase), FPG (continuous, per 1-unit increase), clinical presentation (unstable angina pectoris as reference), coronary artery disease (CAD) severity (one-vessel disease as reference), lesions > 20 mm long (with or without), restenotic lesions (with or without), use of drug-coated balloon (yes or no), complete revascularization (yes or no), and use of insulin at discharge (yes or no). In the multivariate model for the key secondary endpoint, the following confounding variables were chosen: age (continuous, per 1-year increase), BMI (continuous, per 1-unit increase), hypertension (with or without), past PCI (with or without), peripheral artery disease (with or without), cardiac failure (with or without), LVEF (continuous, per 1% increase), serum creatinine (continuous, per 1-unit increase), LDL-C (continuous, per 1-unit increase), glycated haemoglobin (continuous, per 1% increase), clinical presentation (unstable angina pectoris as reference), CAD severity (one-vessel disease as reference), lesions > 20 mm long (with or without), complete revascularization (yes or no), and use of angiotensin-converting enzyme inhibitors/angiotensin II receptor blockers (ACEIs/ARBs) at discharge (yes or no). Interaction was tested with a likelihood ratio test, and the proportional hazard assumption was tested by demonstrating no importance of variables multiplied by time as time-dependent variables. Post hoc subgroup analyses stratified by age (< 60 versus ≥60 years), sex (female versus male), BMI (< 28 versus ≥28 kg/m^2^), LDL-C (≤1.8 versus > 1.8 mmol/L), hypertension (yes versus no), and clinical presentation (non-ST versus ST segment elevation ACS) were used to determine the consistency of the prognostic impacts of the AIP as a continuous variable for the primary endpoint. SPSS version 24.0 (IBM Corp., Armonk, New York, US) was used for analyses. A 2-sided *P*-value < 0.05 was considered significant.

## Results

A total of 798 T2DM patients with ACS undergoing PCI were included in the present analysis. The mean age of these patients was 61 ± 10 years, and male patients accounted for 72.7%. The baseline clinical and laboratory characteristics of the study population according to the AIP quartiles are listed in Table 1. Patients with higher AIP values tended to be younger, were predominantly male, had higher rates of current smoking and dyslipidaemia, and had lower rates of never smoking and diagnosis with unstable angina pectoris. Patients with higher AIP values were more likely than patients with lower AIP values to have higher levels of BMI, serum creatinine, uric acid, total cholesterol, LDL-C, triglycerides, and FPG but lower levels of HDL-C. The use of medications, angiographic findings, and procedural results of the study population according to the AIP quartiles are summarized in Table 2. With the exception of β-blockers, medications before admission did not differ across the different AIP groups. With the exception of P2Y12 inhibitors, ACEIs/ARBs, and oral antidiabetic agents, medications at discharge were similar among the different AIP groups. Patients who had higher AIP values were more likely to receive ACEIs/ARBs at discharge. The proportions of left main/three-vessel disease and two-vessel disease were different among the different AIP groups. Patients with higher AIP values tended to have a higher rate of chronic total occlusions and a lower rate of heavy calcification lesions. The proportions of left circumflex artery and right coronary artery interventions were different among the different AIP groups. Patients with higher AIP values tended to have a lower rate of complete revascularization.

**Table 1:** Baseline clinical and laboratory characteristics of the study population according to the AIP quartiles

Variable	Q1	Q2	Q3	Q4	*P* value						
	*n* = 199	*n* = 200	*n* = 200	*n* = 199	Overall	Q2 vs. Q1 *	Q3 vs. Q1 *	Q3 vs. Q2 *	Q4 vs. Q1 *	Q4 vs. Q2 *	Q4 vs. Q3 *
**Demographics**											
Age (years)	63 ± 8	63 ± 10	61 ± 9	58 ± 12	< 0.001	0.750	0.051	0.498	< 0.001	< 0.001	0.007
Male sex, n (%)	125 (62.8)	146 (73.0)	153 (76.5)	156 (78.4)	0.002	0.029	0.003	0.420	0.001	0.209	0.651
**Clinical values (on admission)**											
BMI (kg/m^2^)	25.3 ± 3.1	25.4 ± 2.8	26.1 ± 3.1	27.0 ± 3.5	< 0.001	0.997	0.081	0.129	< 0.001	< 0.001	0.028
SBP (mm Hg)	133 ± 18	133 ± 17	130 ± 16	131 ± 16	0.116	–	–	–	–	–	–
DBP (mm Hg)	75 ± 11	76 ± 10	75 ± 10	77 ± 11	0.203	–	–	–	–	–	–
**Risk factors**											
Smoking status											
Current smoking, n (%)	60 (30.2)	77 (38.5)	76 (38.0)	111 (55.8)	< 0.001	0.079	0.098	0.918	< 0.001	0.001	< 0.001
Former smoking, n (%)	33 (16.6)	33 (16.5)	38 (19.0)	26 (13.1)	0.455	–	–	–	–	–	–
Never smoking, n (%)	106 (53.3)	90 (45.0)	86 (43.0)	62 (31.2)	< 0.001	0.099	0.040	0.687	< 0.001	0.004	0.014
Chronically daily drinking, n (%)	14 (7.0)	18 (9.0)	26 (13.0)	21 (10.6)	0.234	–	–	–	–	–	–
Family history of CHD, n (%)	54 (27.1)	60 (30.0)	69 (34.5)	56 (28.1)	0.386	–	–	–	–	–	–
Hypertension, n (%)	138 (69.3)	137 (68.5)	141 (70.5)	130 (65.3)	0.715	–	–	–	–	–	–
Dyslipidaemia, n (%)	107 (53.8)	174 (87.0)	192 (96.0)	195 (98.0)	< 0.001	< 0.001	< 0.001	0.001	< 0.001	< 0.001	0.245
Previous MI, n (%)	36 (18.1)	43 (21.5)	40 (20.0)	51 (25.6)	0.301	–	–	–	–	–	–
Past PCI, n (%)	47 (23.6)	50 (25.0)	50 (25.0)	44 (22.1)	0.891	–	–	–	–	–	–
Previous ischemic stroke or TIA, n (%)	13 (6.5)	13 (6.5)	17 (8.5)	8 (4.0)	0.338	–	–	–	–	–	–
PAD, n (%)	27 (13.6)	29 (14.5)	31 (15.5)	33 (16.6)	0.853	–	–	–	–	–	–
Cardiac failure, n (%)	12 (6.0)	13 (6.5)	23 (11.5)	19 (9.5)	0.156	–	–	–	–	–	–
LVEF (%)	64 (60–67)	64 (60–68)	65 (60–68)	65 (59–68)	0.963	–	–	–	–	–	–
**Clinical presentation**											
UAP, n (%)	171 (85.9)	153 (76.5)	150 (75.0)	153 (76.9)	0.033	0.016	0.006	0.726	0.020	0.928	0.660
NSTEMI, n (%)	19 (9.5)	28 (14.0)	28 (14.5)	25 (12.6)	0.493	–	–	–	–	–	–
STEMI, n (%)	9 (4.5)	19 (9.5)	22 (11.0)	21 (10.6)	0.088	–	–	–	–	–	–
**Laboratory measurements (fasting state)**											
SCr (μmol/L)	66.5 (59.3–73.6)	69.2 (61.9–80.3)	70.3 (62.2–81.3)	71.5 (63.2–82.2)	< 0.001	0.041	0.023	1.000	< 0.001	0.966	1.000
UA (μmol/L)	303.2 (271.7–347.2)	321.3 (279.6–373.0)	323.8 (282.5–386.0)	368.5 (307.0–410.3)	< 0.001	0.376	0.011	1.000	< 0.001	< 0.001	< 0.001
TC (mmol/L)	3.90 ± 0.96	4.06 ± 1.01	4.10 ± 0.99	4.47 ± 1.03	< 0.001	0.341	0.179	0.984	< 0.001	< 0.001	0.002
LDL-C (mmol/L)	2.23 ± 0.85	2.48 ± 0.84	2.48 ± 0.77	2.53 ± 0.76	0.001	0.011	0.012	1.000	0.001	0.926	0.916
HDL-C (mmol/L)	1.23 ± 0.22	1.04 ± 0.17	0.95 ± 0.17	0.87 ± 0.14	< 0.001	< 0.001	< 0.001	< 0.001	< 0.001	< 0.001	< 0.001
Triglycerides (mmol/L)	0.88 (0.72–1.00)	1.31 (1.18–1.48)	1.71 (1.51–1.99)	2.66 (2.25–3.38)	< 0.001	< 0.001	< 0.001	< 0.001	< 0.001	< 0.001	< 0.001
FPG (mmol/L)	6.80 (6.12–8.23)	7.17 (6.32–8.25)	6.80 (5.91–7.88)	7.65 (6.61–8.49)	< 0.001	0.466	1.000	0.070	0.003	0.531	< 0.001
Glycated haemoglobin (%)	7.0 (6.6–8.1)	7.3 (6.7–8.2)	7.2 (6.6–8.1)	7.3 (6.7–8.0)	0.409	–	–	–	–	–	–
AIP	−0.1581 ± 0.1373	0.1029 ± 0.0482	0.2631 ± 0.0504	0.5272 ± 0.1773	< 0.001	< 0.001	< 0.001	< 0.001	< 0.001	< 0.001	< 0.001

* *P* < 0.0083 is considered statistically significant for post hoc multiple comparisons of categorical variables between groups

*AIP* Indicates atherogenic index of plasma; *BMI* Body mass index; *SBP* Systolic blood pressure; *DBP* Diastolic blood pressure; *CHD* Coronary heart disease; *MI* Myocardial infarction; *PCI* Percutaneous coronary intervention; *TIA* Transient ischemic attack; *PAD* Peripheral artery disease; *LVEF* Left ventricular ejection fraction; *UAP* Unstable angina pectoris; *NSTEMI* Non ST-segment elevation myocardial infarction; *STEMI* ST-segment elevation myocardial infarction; *SCr* Serum creatinine; *UA* Uric acid; *TC* Total cholesterol; *LDL-C* Low-density lipoprotein-cholesterol; *HDL-C* High-density lipoprotein-cholesterol; *FPG* Fasting plasma glucose

**Table 2:** Use of medications, agiographic findings, and procedural results of the study population according to the AIP quartiles

Variable	Q1	Q2	Q3	Q4	*P* value						
	*n* = 199	*n* = 200	*n* = 200	*n* = 199	Overall	Q2 vs. Q1 *	Q3 vs. Q1 *	Q3 vs. Q2 *	Q4 vs. Q1 *	Q4 vs. Q2 *	Q4 vs. Q3 *
**Medications before admission**											
Aspirin, n (%)	145 (72.9)	149 (74.5)	154 (77.0)	146 (73.4)	0.784	–	–	–	–	–	–
P2Y12 inhibitors, n (%)	81 (40.7)	81 (40.5)	74 (37.0)	80 (40.2)	0.858	–	–	–	–	–	–
Statins, n (%)	150 (75.4)	143 (71.5)	155 (77.5)	142 (71.4)	0.418	–	–	–	–	–	–
ACEIs/ARBs, n (%)	55 (27.6)	65 (32.5)	72 (36.0)	69 (34.7)	0.300	–	–	–	–	–	–
β-blockers, n (%)	70 (35.2)	79 (39.5)	93 (46.5)	63 (31.7)	0.016	0.372	0.021	0.157	0.457	0.102	0.002
Insulin, n (%)	75 (37.7)	73 (36.5)	79 (39.5)	63 (31.7)	0.406	–	–	–	–	–	–
Oral antidiabetic agents, n (%)	89 (44.7)	98 (49.0)	110 (55.0)	90 (45.2)	0.145	–	–	–	–	–	–
**Intraoperative anticoagulants**											
Unfractionated heparin, n (%)	168 (84.4)	170 (85.0)	154 (77.0)	156 (78.4)	0.086	–	–	–	–	–	–
LMWH, n (%)	6 (3.0)	7 (3.5)	16 (8.0)	10 (5.0)	0.089	–	–	–	–	–	–
Bivalirudin, n (%)	25 (12.6)	23 (11.5)	30 (15.0)	33 (16.6)	0.448	–	–	–	–	–	–
**Perioperative medications**											
Aspirin, n (%)	196 (98.5)	198 (99.0)	200 (100.0)	196 (98.5)	0.308	–	–	–	–	–	–
P2Y12 inhibitors, n (%)	199 (100.0)	200 (100.0)	200 (100.0)	199 (100.0)	–	–	–	–	–	–	–
GP IIb/IIIa receptor antagonist, n (%)	29 (14.6)	38 (19.0)	45 (22.5)	37 (18.6)	0.246	–	–	–	–	–	–
**Medications at discharge**											
Aspirin, n (%)	196 (98.5)	198 (99.0)	200 (100.0)	196 (98.5)	0.308	–	–	–	–	–	–
Cilostazol, n (%)	3 (1.5)	2 (1.0)	1 (0.5)	4 (2.0)	0.473	–	–	–	–	–	–
Clopidogrel, n (%)	174 (87.4)	183 (91.5)	178 (89.0)	190 (95.5)	0.031	0.186	0.628	0.399	0.004	0.108	0.016
Ticagrelor, n (%)	25 (12.6)	17 (8.5)	22 (11.0)	9 (4.5)	0.031	0.186	0.628	0.399	0.004	0.108	0.016
Statins, n (%)	199 (100.0)	200 (100.0)	200 (100.0)	199 (100.0)	–	–	–	–	–	–	–
ACEIs/ARBs, n (%)	81 (40.7)	93 (46.5)	105 (52.5)	121 (60.8)	0.001	0.243	0.018	0.230	< 0.001	0.004	0.094
β-blockers, n (%)	138 (69.3)	147 (73.5)	155 (77.5)	136 (68.3)	0.155	–	–	–	–	–	–
Insulin, n (%)	65 (32.7)	76 (38.0)	72 (36.0)	57 (28.6)	0.213	–	–	–	–	–	–
Oral antidiabetic agents, n (%)	87 (43.7)	120 (60.0)	119 (59.5)	105 (52.8)	0.003	0.001	0.002	0.919	0.071	0.145	0.175
**Angiographic findings**											
One-vessel disease, n (%)	22 (11.1)	15 (7.5)	18 (9.0)	25 (12.6)	0.347	–	–	–	–	–	–
Two-vessel disease, n (%)	68 (34.2)	41 (20.5)	42 (21.0)	46 (23.1)	0.004	0.002	0.003	0.902	0.015	0.527	0.610
LM/three-vessel disease, n (%)	109 (54.8)	144 (72.0)	140 (70.0)	128 (64.3)	0.001	< 0.001	0.002	0.659	0.052	0.100	0.227
Proximal LAD stenosis, n (%)	94(47.2)	110 (55.0)	97 (48.5)	100 (50.3)	0.428	–	–	–	–	–	–
Restenotic lesions, n (%)	26 (13.1)	26 (13.0)	30 (15.0)	29 (14.6)	0.913	–	–	–	–	–	–
Trifurcation or bifurcation lesions, n (%)	155 (77.9)	162 (81.0)	151 (75.5)	148 (74.4)	0.403	–	–	–	–	–	–
Chronic total occlusions, n (%)	36 (18.1)	46 (23.0)	44 (22.0)	57 (28.6)	0.093	–	–	–	–	–	–
Thrombus lesions, n (%)	7 (3.5)	14 (7.0)	12 (6.0)	12 (6.0)	0.478	–	–	–	–	–	–
Heavy calcification lesions, n (%)	75 (37.7)	69 (34.5)	73 (36.5)	48 (24.1)	0.016	0.507	0.806	0.676	0.003	0.023	0.007
Lesions > 20 mm long, n (%)	108 (54.3)	112 (56.0)	122 (61.0)	117 (58.8)	0.537	–	–	–	–	–	–
**Procedural results**											
Target vessel territory											
LM, n (%)	13 (6.5)	16 (8.0)	9 (4.5)	18 (9.0)	0.313	–	–	–	–	–	–
LAD, n (%)	104 (52.3)	97 (48.5)	100 (50.0)	99 (49.7)	0.899	–	–	–	–	–	–
LCX, n (%)	64 (32.2)	69 (34.5)	42 (21.0)	48 (24.1)	0.007	0.620	0.012	0.003	0.075	0.023	0.456
RCA, n (%)	69 (34.7)	82 (41.0)	100 (50.0)	74 (37.2)	0.011	0.193	0.002	0.071	0.601	0.435	0.010
DES use, n (%)	174 (87.4)	172 (86.0)	161 (80.5)	159 (79.9)	0.097	–	–	–	–	–	–
BRS use, n (%)	6 (3.0)	7 (3.5)	8 (4.0)	11 (5.5)	0.607	–	–	–	–	–	–
DCB use, n (%)	12 (6.0)	11 (5.5)	19 (9.5)	14 (7.0)	0.409	–	–	–	–	–	–
Complete revascularization, n (%)	136 (68.3)	115 (57.5)	110 (55.0)	107 (53.8)	0.012	0.025	0.006	0.614	0.003	0.453	0.805

* P < 0.0083 is considered statistically significant for post hoc multiple comparisons of categorical variables between groups

*AIP* Indicates atherogenic index of plasma; *ACEIs* Angiotensin converting enzyme inhibitors; *ARBs* Angiotensin II receptor blockers; *LM* Left-main artery; *LAD* Left anterior descending artery; *LCX* Left circumflex artery; *RCA* Right coronary artery; *DES* Drug-eluting stent; *BRS* Bioresorbable scaffold; *DCB* Drug-coated balloon

Patients were followed up for a median of 927 days (interquartile range, 774 to 1109 days). During the period, 198 patients developed at least one adverse cardiovascular event, which was found in 33 (16.6%) patients from the Q1 group, 45 (22.5%) from the Q2 group, 54 (27.0%) from the Q3 group, and 66 (33.2%) from the Q4 group. Of the 198 patients with at least one adverse cardiovascular event, 20 died (18 died from cardiovascular causes, and 2 died from non-cardiovascular causes), 24 developed non-fatal spontaneous MI, 17 developed non-fatal ischaemic stroke, and 180 underwent unplanned repeat revascularization; among these patients, 33 had two, 2 had three, and 2 had four adverse cardiovascular events.

**Fig. 1:**
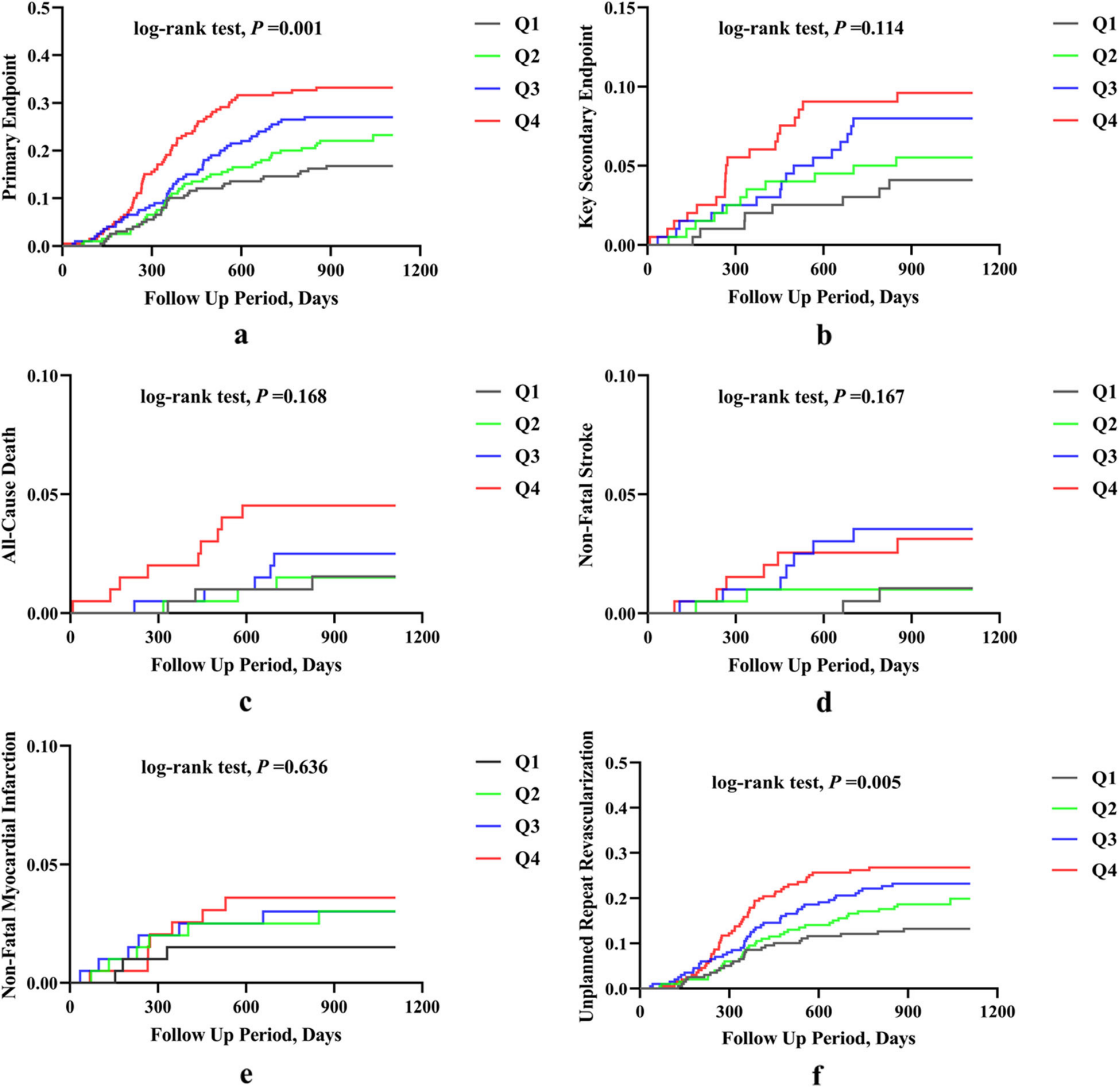
Kaplan-Meier curves for the primary and key secondary endpoints and each component event of the primary endpoint across the AIP quartiles. The primary endpoint was defined as a composite of death from any cause, non-fatal spontaneous myocardial infarction, non-fatal ischaemic stroke, and unplanned repeat revascularization. The key secondary endpoint was a composite of cardiovascular death, non-fatal spontaneous myocardial infarction, and non-fatal ischaemic stroke

Kaplan-Meier analyses revealed a significantly higher incidence of the primary endpoint (log-rank test, *P* = 0.001; Fig. 1a) and a marginally but non-significantly higher incidence of the key secondary endpoint (log-rank test, *P* = 0.114; Fig. 1b) in patients with higher AIP values. The difference in the incidence of the primary endpoint was due mainly to an increase in unplanned repeat revascularization (log-rank test, *P* = 0.005; Fig. 1f) across the AIP quartiles. However, the incidence of death from any cause (log-rank test, *P* = 0.168; Fig. 1c), cardiovascular death (log-rank test, *P* = 0.459), non-fatal ischaemic stroke (log-rank test, *P* = 0.167; Fig. 1d), and non-fatal spontaneous MI (log-rank test, *P* = 0.636; Fig. 1e) did not differ among the AIP quartiles at follow-up.

Table 3 shows univariate and multivariate Cox proportional hazards analyses for the primary endpoint at follow-up. A multivariate Cox proportional hazards analysis, with Q1 as a reference, showed that the AIP for Q4 had a significantly increased HR (2.249, 95% CI 1.438–3.517) for the incidence of the primary endpoint. Moreover, the AIP used as a continuous variable was independently predictive of the primary endpoint (HR, 2.684; 95% CI, 1.585–4.546; *P* < 0.001). Table 4 shows univariate and multivariate Cox proportional hazards analyses for the key secondary endpoint at follow-up. A multivariate Cox proportional hazards analysis, with Q1 as a reference, showed that the AIP for Q4 had a significantly increased HR (2.571, 95% CI 1.027–6.440) for the incidence of the key secondary endpoint. Moreover, the AIP used as a continuous variable was independently predictive of the key secondary endpoint (HR, 3.160; 95% CI, 1.040–9.600; *P* = 0.042).

**Table 3:** Relationship between the incidence of the primary endpoint and the AIP expressed as a categorical variable

Variables	Univariate analysis HR (95% CI)	*P*-value	Multivariate analysis HR (95% CI)	*P*-value
AIP quartiles				
Q1	Reference		Reference	
Q2	1.376 (0.878–2.156)	0.164	1.167 (0.733–1.857)	0.516
Q3	1.710 (1.109–2.636)	0.015	1.570 (0.995–2.478)	0.053
Q4	2.265 (1.491–3.440)	< 0.001	2.249 (1.438–3.517)	< 0.001
Age	1.005 (0.991–1.019)	0.472	0.996 (0.980–1.012)	0.642
BMI	0.959 (0.916–1.005)	0.078	0.922 (0.876–0.970)	0.002
Hypertension	1.068 (0.789–1.447)	0.669	1.241 (0.890–1.729)	0.203
Previous MI	1.492 (1.095–2.033)	0.011	0.781 (0.532–1.145)	0.205
Past PCI	1.723 (1.285–2.310)	< 0.001	1.263 (0.734–2.080)	0.425
PAD	2.511 (1.836–3.435)	< 0.001	1.717 (1.192–2.473)	0.004
Cardiac failure	1.951 (1.308–2.909)	0.001	1.165 (0.677–2.004)	0.581
LVEF	0.974 (0.959–0.990)	0.002	0.983 (0.962–1.004)	0.120
SCr	1.013 (1.006–1.021)	0.001	1.010 (1.002–1.019)	0.014
LDL-C	1.078 (0.916–1.269)	0.366	1.028 (0.852–1.239)	0.774
FPG	1.201 (1.132–1.274)	< 0.001	1.156 (1.087–1.230)	< 0.001
Clinical presentation				
UAP	Reference		Reference	
NSTEMI	0.677 (0.416–1.102)	0.117	0.467 (0.275–0.792)	0.005
STEMI	1.098 (0.690–1.747)	0.695	0.850 (0.508–1.424)	0.538
CAD severity				
One-vessel disease	Reference		Reference	
Two-vessel disease	1.756 (0.809–3.810)	0.154	1.230 (0.549–2.755)	0.615
LM/three-vessel disease	3.541 (1.740–7.205)	< 0.001	1.823 (0.858–3.873)	0.118
Restenotic lesions	2.290 (1.658–3.163)	< 0.001	1.667 (0.914–3.040)	0.095
Lesions > 20 mm long	1.984 (1.461–2.695)	< 0.001	1.566 (1.129–2.174)	0.007
DCB use	1.913 (1.228–2.980)	0.004	0.990 (0.571–1.715)	0.970
Complete revascularization	0.467 (0.352–0.619)	< 0.001	0.687 (0.504–0.936)	0.017
Insulin at discharge	1.396 (1.051–1.854)	0.021	1.056 (0.775–1.438)	0.730

Abbreviations as in Tables 1 and 2

**Table 4:** Relationship between the incidence of the key secondary endpoint and the AIP expressed as a categorical variable

Variables	Univariate analysis HR (95% CI)	*P*-value	Multivariate analysis HR (95% CI)	*P*-value
AIP quartiles				
Q1	Reference		Reference	
Q2	1.380 (0.555–3.432)	0.488	0.985 (0.362–2.679)	0.976
Q3	2.020 (0.865–4.720)	0.104	1.822 (0.734–4.524)	0.196
Q4	2.‘477 (1.085–5.659)	0.031	2.571 (1.027–6.440)	0.044
Age	1.042 (1.013–1.072)	0.004	1.016 (0.984–1.048)	0.331
BMI	0.915 (0.834–1.004)	0.062	0.861 (0.775–0.956)	0.005
Hypertension	1.333 (0.725–2.450)	0.354	1.303 (0.664–2.558)	0.442
Past PCI	1.904 (1.096–3.309)	0.022	2.124 (1.119–4.030)	0.021
PAD	5.804 (3.402–9.902)	< 0.001	3.068 (1.677–5.613)	< 0.001
Cardiac failure	6.939 (3.993–12.061)	< 0.001	1.870 (0.774–4.517)	0.164
LVEF	0.924 (0.902–0.946)	< 0.001	0.947 (0.912–0.983)	0.004
SCr	1.025 (1.014–1.036)	< 0.001	1.010 (0.997–1.022)	0.123
LDL-C	1.064 (0.774–1.463)	0.702	1.102 (0.731–1.661)	0.644
Glycated haemoglobin	1.090 (0.882–1.347)	0.425	1.243 (0.974–1.587)	0.081
Clinical presentation				
UAP	Reference		Reference	
NSTEMI	1.333 (0.622–2.857)	0.460	0.462 (0.187–1.143)	0.095
STEMI	1.920 (0.896–4.115)	0.094	0.880 (0.375–2.061)	0.768
CAD severity				
One-vessel disease	Reference		Reference	
Two-vessel disease	0.201 (0.037–1.096)	0.064	0.060 (0.009–0.399)	0.004
LM/three-vessel disease	1.910 (0.689–5.296)	0.214	0.476 (0.142–1.593)	0.228
Lesions > 20 mm long	2.681 (1.411–5.029)	0.003	1.776 (0.866–3.640)	0.117
Complete revascularization	0.403 (0.232–0.700)	0.001	0.677 (0.346–1.321)	0.253
ACEIs/ARBs at discharge	2.933 (1.596–5.391)	0.001	1.659 (0.825–3.335)	0.155

Abbreviations as in Tables 1 and 2

**Fig. 2:**
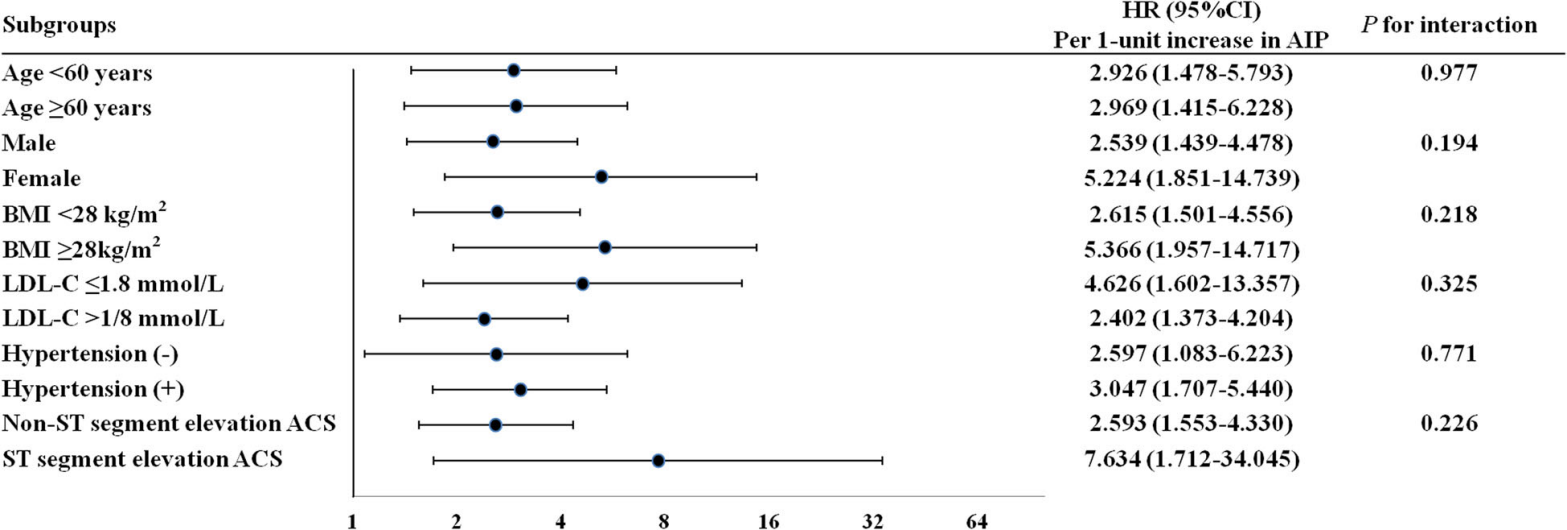
Cox proportional hazards analyses for the primary endpoint at follow-up in the different subgroups. HR was evaluated by per 1-unit increase in the AIP. BMI indicates body mass index; LDL-C, low-density lipoprotein-cholesterol; ACS, acute coronary syndrome; HR, hazard ratio; 95% CI, 95% confidence interval

The predictive impacts of the AIP as a continuous variable for the primary endpoint were further assessed in different subgroups of the study population. Increased AIP value (per 1-unit) was consistently associated with the primary endpoint in different subgroups, including age < 60 versus ≥60 years, female versus male sex, BMI < 28 versus ≥28 kg/m^2^, LDL-C ≤ 1.8 versus > 1.8 mmol/L, with versus without hypertension, and non-ST versus ST segment elevation ACS (Fig. 2).

## Discussion

The main findings of this study were as follows: (1) patients with higher AIP values had a significantly higher probability of adverse cardiovascular events by the log-rank test, and (2) multivariate Cox proportional hazards analyses showed that the AIP was independently and strongly associated with the primary and key secondary endpoints in T2DM and ACS patients who underwent PCI, suggesting that the AIP might have a potential role in early risk stratification of such patients.

Dyslipidaemia plays a crucial role in the pathogenesis and progression of coronary atherosclerosis [37]. Diabetic patients are more likely to develop coronary atherosclerosis than non-diabetic patients, which may be significantly associated with so-called diabetic dyslipidaemia, which consists of elevated plasma concentrations of triglyceride-rich lipoproteins and small dense LDL-C particles and low levels of HDL-C. The different components of diabetic dyslipidaemia are not isolated abnormalities but are closely linked to each other metabolically [12]. Triglycerides and HDL-C are two lipid parameters measured routinely in clinical practice; however, neither is a consistently good proxy for plasma atherogenicity. The AIP, a novel lipid index defined as log_10_ (triglycerides/HDL-C), has been demonstrated to be closely correlated with FER (HDL) and lipoprotein particle size, both of which are directly involved in the pathogenesis and development of atherosclerosis; thus, the AIP is regarded as an excellent indicator of atherosclerosis and can offer a benefit to identify the risk of cardiovascular disease [38].

Multiple cross-sectional studies reported that the AIP was a strong predictor of CAD independent of diabetes [26, 27, 29]. Notably, the Indian Atherosclerosis Research Study revealed that the addition of the AIP and family history to traditional risk factors improved risk discrimination (C-index: from 0.864 to 0.873) in Asian Indians with CAD [22]. Intriguingly, Frohlich J et al. found that the AIP was an independent predictor of angiographically defined CAD only when FER (HDL) was omitted from multivariate analysis, which may be due to a clear internal correlation between the AIP and FER (HDL) [20]. Moreover, Nam JS et al. found that there is a significant correlation between the AIP and the progression of coronary artery calcification measured by using multidetector computed tomography in subjects without cardiovascular disease [31]. Furthermore, in a prospective cohort study including 2676 middle-aged adults followed for 7.8 years, researchers demonstrated that the top quartiles of the AIP predicted significantly age-adjusted incident CAD in both sexes, more strongly in women, after adjustment for C-reactive protein and traditional risk factors [21].

The AIP has been demonstrated to be associated with mortality in elderly patients and dialysis patients. Edwards MK et al. analysed data from the 1999–2006 National Health and Nutrition Examination Survey with follow-up through 2011 to find that the AIP was positively and independently associated with mortality risk and predicted mortality risk better than individual cholesterol risk factors among an older adult population [23]. Bendzala M et al. also found that the AIP was positively associated with the risk of all-cause death in elderly women with hypertension [24]. However, a Korean nationwide prospective cohort study including 1174 incident dialysis patients showed that the AIP had a non-linear relationship with survival; both the highest and the lowest AIP quintiles were independently associated with all-cause mortality, showing a U-shaped association [25].

The predictive value of the AIP has also been explored in ACS patients. Cai G et al. retrospectively enrolled 1478 very young participants (≤35 years of age) undergoing coronary angiography and divided them into two groups: the ACS group (*n* = 1058) and the non-CAD group (*n* = 419). They found that the AIP was independently associated with the presence and severity of ACS in a sex-dependent manner and that the prevalence of ACS, acute MI, and unstable angina pectoris and the Gensini score (a scoring system for evaluating CAD severity) were elevated as AIP quartiles increased [28]. Qin Z et al. retrospectively enrolled 2356 T2DM patients who underwent PCI and followed them for 4 years. They found that the AIP was an independent predictor of major cardiovascular and cerebrovascular adverse events, including cardiac death, MI, repeated revascularization, and stroke, regardless of clinical presentation [30].

The AIP was reported to be positively correlated with serum malondialdehyde levels in menopausal women with cardiovascular disease [39], and malondialdehyde can reflect the status of oxidative stress, which is significantly associated with coronary atherosclerosis [40]. The AIP was found to be associated with epicardial adipose tissue measured by using transthoracic echocardiography or electrocardiogram-gated multidetector computed tomography [41, 42]. Evidence indicates that epicardial adipose tissue directly affects coronary atherosclerosis [43]. The AIP was shown to be directly and independently associated with arterial stiffness in normotensive and never-treated hypertensive subjects [44]. Increased aortic stiffness often results in early wave reflection of the aortic pulse wave, which increases systolic blood pressure but decreases diastolic blood pressure, and these haemodynamic changes impair coronary perfusion, which can promote adverse cardiovascular events [36]. The AIP was demonstrated to be a marker for reduced coronary flow reserve [45], and the latter was positively associated with adverse cardiovascular events. Of note, the AIP comprises HDL-C and triglycerides in its formula. Low HDL-C and high triglyceride levels were shown to be associated with adverse cardiovascular events after ACS, independent of diabetic status [46, 47].

Glucose-lowering regimens including pioglitazone have been shown to be effective in reducing AIP values, thereby reducing cardiovascular risk [48, 49]. Moreover, moderate-to-vigorous physical activity, increased aerobic exercise time, decreased sedentary behaviour, and consequent high levels of cardiorespiratory fitness were reported to be inversely correlated with the AIP, which implies that a healthy lifestyle helps attenuate the risk for cardiovascular disease via AIP improvements [50–54]. As a result, the use of pioglitazone and early initiation of physical activity and aerobic exercise may be important to reduce future cardiovascular risk in T2DM patients with ACS undergoing PCI who have received first-line medication therapy but still have significantly increased AIP values.

### Study strength and limitations

The present study demonstrated a significant association between the AIP and adverse cardiovascular outcomes in T2DM patients with ACS undergoing PCI. Even after adjustment for as many potential confounding variables as possible, there remained an independent association of the AIP with adverse cardiovascular outcomes. To our knowledge, this study is the first to report the prognostic impact of the AIP in T2DM and ACS patients who underwent PCI.

The present study has several limitations. First, this study was a retrospective analysis of a single-centre prospective registry. As in other observational studies, the effects of unmeasured and undetected confounding variables cannot be excluded in this study. Second, the baseline concentrations of triglycerides and HDL-C might be affected by the use of statins before admission and glycaemic control status. However, there were no significant differences among the AIP quartiles with respect to the use of statins before admission and glycated haemoglobin, which can consistently reflect glycaemic control status in diabetic patients. Third, unhealthy lifestyle and obstructive sleep apnea are associated with dyslipidemia and the development of cardiovascular disease. However, information on lifestyle (such as diet, exercise, etc.) and obstructive sleep apnea was not recorded and considered in this study. Fourth, follow-up data were obtained merely via telephone; however, the authenticity of adverse events was confirmed by reviewing corresponding medical records. Finally, all study patients were Chinese. While ethnic homogeneity might be considered an advantage, the findings derived from the present study may not be extrapolated to other ethnic groups without caution.

## Conclusions

A higher AIP value on admission was independently and strongly associated with adverse cardiovascular events in T2DM patients with ACS undergoing PCI, suggesting that the AIP might have a potential role in the early risk stratification of such patients. Medical management optimization according to the AIP could result in a reduced risk of subsequent cardiovascular events.

## Data Availability

The data, analytic methods, and study materials will not be made available to other researchers for purposes of reproducing the results or replicating the procedure.

## Abbreviations

ACEIs/ARBs: Angiotensin-converting enzyme inhibitors/angiotensin II receptor blockers
ACS: Acute coronary syndrome
AIP: Atherogenic index of plasma
BMI: Body mass index
CAD: Coronary artery disease
CI: Confidence interval
FER (HDL): Cholesterol esterification rates in apoB-lipoprotein-depleted plasma
FPG: Fasting plasma glucose
HDL-C: High-density lipoprotein-cholesterol
HR: Hazard ratio
LDL-C: Low-density lipoprotein-cholesterol
LVEF: Left ventricular ejection fraction
MI: Myocardial infarction
PCI: Percutaneous coronary intervention
T2DM: Type 2 diabetes mellitus
